# Mediators of the relation between war experiences and suicidal ideation among former child soldiers in Northern Uganda: the WAYS study

**DOI:** 10.1186/s12888-014-0271-2

**Published:** 2014-09-24

**Authors:** Kennedy Amone-P’Olak, Tlholego Molemane Lekhutlile, Richard Meiser-Stedman, Emilio Ovuga

**Affiliations:** Department of Psychology, University of Botswana, Private Bag UB 00705, Gaborone, Botswana; MRC Cognition and Brain Sciences Unit, Cambridge, UK; Department of Psychiatry and Mental Health, Gulu University, P O Box 166, Gulu, Uganda

**Keywords:** War experiences, Suicidal ideation, Former child soldiers, Northern Uganda

## Abstract

**Background:**

Globally, suicide is a public health burden especially in the aftermath of war. Understanding the processes that define the path from previous war experiences (WE) to current suicidal ideation (SI) is crucial for defining opportunities for interventions. We assessed the extent to which different types of previous WE predict current SI and whether post-war hardships and depression mediate the relations between WE and SI among former child soldiers (FCS) in Northern Uganda.

**Methods:**

We performed cross-sectional analyses with a sample of 539 FCS (61% male) participating in an on-going longitudinal study. The influence of various types of previous WE on current SI and mediation by post-war hardships and depression were assessed by regression analyses.

**Results:**

The following types of war experiences: “witnessing violence”, “direct personal harm”, “deaths”, “Involvement in hostilities”, “sexual abuse” and “general war experiences” significantly predicted current SI in a univariable analyses whereas “direct personal harm”, “involvement in hostilities”, and “sexual abuse” independently predicted current SI in a multivariable analyses. General WE were linked to SI (β = 0.18 (95% CI 0.10 to 0.25)) through post-war hardships (accounting for 69% of the variance in their relationship) and through depression/anxiety (β = 0.17 (95% CI 0.12 to 0.22)) accounting for 65% of the variance in their relationship. The direct relationship between previous WE and current SI reduced but remained marginally significant (β = .08, CI: (.01, .17) for depression/anxiety but not for post-war hardships (β = .09, CI: (−.03, .20).

**Conclusion:**

Types of WE should be examined when assessing risks for SI. Interventions to reduce SI should aim to alleviate post-war hardships and treat depression/anxiety.

## Background

Globally, suicide is a serious public health problem and it is among three leading causes of death in 15 – 44 age bracket [1]. Annually, it is estimated that one million people die as a result of suicide [1-3] and it is projected that suicide will account for 2.4% of the total death burden by 2020 [4]. In Africa, the annual incidence rate is estimated to be 3.2 per 100,000 people [5]. Suicidal behaviour is a process and it evolves through a continuum ranging from ideation (thoughts about suicide) to attempts (varying degrees of attempts to end one’s life), to completed suicide, i.e. successfully taking one’s life [6]. Previous studies on suicide indicate that cases of completed suicides are higher in males while suicidal ideation (SI), attempted suicides, and suicidal threats are higher among females [7-9]. Focusing on SI is important for identifying those at risk of suicide attempts and completion. In addition, preventive strategies have been suggested to be successful at this stage [2].

There are numerous risk factors associated with SI. These risk factors include mental health problems (e.g. depression), behavioural problems (e.g. drug and substance abuse), terminal illnesses and health conditions such as HIV/AIDS, exposure to extreme poverty, and previous traumatic life experiences such as war events [2,6,10-13]. The current study explored factors that account for influence of war experiences (WE) on current SI among former child soldiers in Northern Uganda. Northern Uganda endured a long and brutal war for about two decades in which thousands of people were killed, the local economy ruined, and individual lives wrecked, thousands of children abducted, and over 90% of the population in war-affected region displaced [14-17]. Studies on suicide in Northern Uganda in the aftermath of the war have associated suicidal behaviours with alcohol [18], negative life events [19], and despair [20].

Previous WE may lead to SI in at least four or more pathways. First it can lead to mental health problems such as depression and PTSD which are known risk factors for suicide [21]. Second, previous WE have also been associated with physical health problems such as loss of mobility, bomb fragments and bullets lodged in the bodies of war survivors and other serious injuries that require life-long support that are often absent in the aftermath of war and which may lead one to contemplate suicide [22]. Third, disabilities and mental health problems associated with previous WE are often associated with alcohol and substance abuse, divorce, domestic violence, all of which are risk factors for suicidal behaviours [23]. Fourth, previous participation in active combat, especially killing, has been suggested to be closely related to SI [24]. For example, suicide is a common cause of death in United States military and accounted for substantial mortality in the aftermath of the Iraq conflict during the Operation Desert Storm [25]. Lastly, the post-war environment is fraught with stressors such as endemic poverty, unemployment and lack of opportunities for livelihoods, financial difficulties, conflicts, and disempowerment, all of which are risk factors for suicidal behaviours [22,26-29]. Among war-affected populations, former child soldiers (FCS) also experience difficulties in controlling their aggressive behaviour and it is suggested that they have fewer skills to handle such stressors without the use of violence [30]. Similarly, previous WE are also known risk factors for depression/anxiety, PTSD, and substance abuse, which in turn, are risk factors for SI in war-affected populations [31-39]. FCS have typically been exposed to many of these risk factors, but how their WE impact on their well-being and functioning is poorly understood. In particular, the role of mental health (particularly depression) in mediating the relationship between previous WE and current SI among FCS has not been studied. Mediation analyses is appropriate for this study because the WAYS study assessed previous war experiences (more than six years ago), post-war difficulties and depression in the past year and current Suicidal Ideation. Consequently, the temporal order of events in this study (prerequisite for mediation analyses) meets the condition for a mediation analyses.

Most previous studies have examined the associations of general exposure to WE with subsequent SI without considering the possibility that particular types of previous WE may put survivors at more risks of SI [31-34]. One such study found that killing during combat predicted SI [33]. In a previous study based on the same population and data, exposure to previous WE was associated with post-war hardships and post-war hardships, in turn, was linked to mental health problems including depression and anxiety [35]. It is therefore possible that post-war hardships and depression may mediate the associations between previous WE and current SI. This study uses data from an on-going longitudinal cohort (***W***ar-***A***ffected ***Y***ouths ***S***urvey - WAYS) study in northern Uganda [35]. Using cross-sectional analyses of a longitudinal study in Northern Uganda, we report on an analysis of data using a robust measure of demographic characteristics, previous WE, post-war hardships and current SI. The objectives of our study were threefold: 1) to assess the incidences of SI in our sample, 2) to investigate the extent to which different types of previous WE predict current SI, 3) to quantify, through our analyses, the extent to which the relation between previous WE and current SI are mediated by current post-war hardships and depression.

## Methods

### Sample

The WAYS study utilised a cohort study design recruiting FCS from five districts that were most affected by the war in Northern Uganda (Gulu, Amuru, Nwoya, Pader, and Kitgum). These districts consist of smaller geographically defined administrative units (hereafter referred to as sub-counties) where the FCS were members of particular groups formed for easy access and social support. A cluster sampling technique was used to recruit participants in the study from a list compiled by UNICEF. The list compiled by UNICEF was previously used to allow formerly abducted children get assistance from NGOs, including UNICEF. Given the importance of being on this list and UNICEF being a credible international UN body, it is assumed that the list is comprehensive and fairly accurate. Young people on this list were eligible to enter the study if they met the following inclusion criteria: 1) history of abduction by rebels, 2) lived in rebel captivity for at least 6 months; and 3) aged 18–25 years. Those who met the above inclusion criteria were invited through their local council leaders to participate in the study. In total 650 formerly abducted children were invited to participate and data was collected from 539 of them representing 83%. The cohort profile is described in detail elsewhere [35]. Baseline data was collected between June to September 2011. The data presented in this paper are drawn from the baseline data.

### Data collection

The interviewers conducting fieldwork for the WAYS study were all university graduates who had been extensively trained in data collection and interviewing skills, briefed on the study background, and detailed interview content. All the interviewers were fluent in speaking and writing the native language of the participants. The interviewers visited the participants in their homes or nearby trading centres or community halls to conduct semi-structured face-to-face interviews and to administer questionnaires covering a wide range of topics. Information sought in the questionnaire included, among others, demographic characteristics, their previous WE, mental health problems and post-war hardships in the past year, and current SI. A Clinical Psychiatric Officer was available on sight to handle mental health emergencies and to make referrals to the Regional Referral Hospital in case of a mental health emergency such as severe depression or conduct problem with a potential for harm. Ethical approval for this study was obtained from Gulu University’s Institutional Review Board, an affiliate of Uganda National Council for Science and Technology which oversees all research activities in Uganda.

Written informed consent was obtained from all participants in accordance with ethical guidelines and approvals.

### Measures

Finding appropriate and standardised measures to assess of mental health outcomes in non-western settings with differences in culture and absence of culturally specific standardised measures is a challenge. Therefore, we used both standardised and locally derived measures.

#### War experiences

To assess individual exposures to different types of WE, we used items from the UNICEF B&H (B&H – Bosnia-Herzegovina) Post-war Screening Survey [40]. The questionnaire was adapted to better capture the context of the war in Northern Uganda; for example, an item on sexual assault and/or abuse was added. Consequently, the adapted WE measure consisted of 52 items from which 12 types of war events were derived. The 12 types of WE were: personal harm (6 items, e.g. serious injuries), witnessing general war violence (11 items, e.g., massacres or raids on villages), sexual abuse (1 item), and involvement in hostilities (2 items, e.g. did you fight in the army or warring faction?). Other types of WE included: separation (2 items), Deaths (7 items, e.g. deaths of parents, siblings, or extended family members), material loss (4 items), physical threat to self (5 items), harm to loved ones (4 items), physical threat to relatives or loved ones (4 items), displacement (5 items), and drug and substance abuse (1 item). The 52 items were summed up for occurrence to generate general exposure to WE. WE were simply binary coded for occurrence (1) versus absence (0). Age at abduction (in months and years) and duration with the fighting forces were self-reported.

#### Suicidal ideation

SI was assessed by a statement: “I think about suicide” and the participants’ responses were scored on a 0–3 scale, ranging from 0 = not at all, 1 = sometimes, 2 = many times, and 3 = all the time. Because a similar item on SI was also present in the depression scale and to ensure that the item was not driving the associations, a sensitivity analysis was conducted without the item on the depression scale.

#### Depression/anxiety

Depression/anxiety is a subscale of the Acholi Psychosocial Assessment Instrument (APAI). APAI is a 40-item field-based self-report questionnaire previously developed for use in Northern Uganda [41]. The measure comprises: depression/anxiety, conduct problems, pro-social behaviours, and somatic complaints without medical cause. In APAI, depression and anxiety (18 items) were combined into one scale. Previous studies also showed a strong overlap among items in the depression and anxiety subscales [42]. The depression/anxiety scale was represented by a set of questions that inquire about specific behaviours particular to depression/anxiety such as “I do not sleep at night”, “I have a lot of thoughts”, etc. For each question, responses were scored on a 0–3 scale, ranging from 0 = never, 1 = rarely, 2 = sometimes, and 3 = always. In previous studies, the Cronbach’s alpha was .70 for the depression subscale [41]. In this study the Cronbach’s alphas were .89 for the depression/anxiety subscale.

#### Post-war hardships

The UNICEF B&H Post-war Screening Survey questionnaire consists of 26 items on difficulties experienced during the past six months [40]. The items included housing and economic difficulties (e.g. “During the past six months, did you lack money for basic necessities like soap, salt, or sugar?”). Each question was binary coded for presence (1) versus absence (0) (range 0 to 26). In this study the Kuder-Richardson coefficient of reliability (KR20) is .83.

### Statistical analyses

The demographic characteristics of the respondents in this study were described and tabulated. Next, the relationships among variables in this study were computed and the correlations coefficients tabulated. The composite general war exposure (WE) was not normally distributed and was transformed by computing its square roots before inclusion into the analyses. In addition, given that many of the respondents experienced multiple WE, the potential for multi-collinearity was assessed as the variance inflation factor (VIF) [43]. A VIF >10 indicates serious multi-collinearity and values >4.0 may be a reason for concern. To determine whether various types of previous WE predict current SI differently, we performed univariable regression analyses where the various types of previous WE were regressed on current SI each at a time and the results presented in a table. To quantify the extent to which the various types of previous WE independently predict current SI, all the types of WE that significantly predicted SI in the univariable model (except general exposure to WE) were all included in the multivariable model simultaneously. To further explain the relationship between general WE and SI, two mediation analyses were performed with post-war hardships and depression/anxiety as mediators in the framework of Baron and Kenny [44]. First, to establish whether post-war stressors explain the relationship between WE and SI, a mediation model was performed. Second, the same analysis was conducted with depression/anxiety as a mediator. For both univariable and multivariable regression models, we standardized the predictor variables to a mean of zero and standard deviation of 1 (Z scores). Because gender was significantly correlated with depression/anxiety and SI, it was considered a confounder and its effect was adjusted for by including it in the models. Duration in captivity and age at abduction did not significantly predict the outcome variables in preliminary analyses and were subsequently dropped from further analyses. In addition, the current study employed cluster sampling to recruit participants in the study. Given the potential for variation by sub-counties from which the participants were sampled, we accounted for clustering by including it in our analyses. Except for SI and gender, WE, post-war hardships, and depression/anxiety were analysed as continuous variables. All the statistical analyses were carried out in STATA OCLA version 12.

## Results

The demographic characteristics and descriptive statistics of the study variables are given in Table 1. Among the participants, 71 (13.17%), 39 (7.24%), and 19 (3.53%) reported SI sometimes, many times, and all the time, respectively. In totoal, 129 (23.93%) reported that they have suicidal thoughts sometimes, many times or all the time. Previous WE significantly correlated with current SI, symptoms of depression/anxiety, and post-war hardships. Current SI significantly correlated with sex, symptoms of depression/anxiety, post-war hardships, and previous WE. Those who reported SI significantly differed from those who did not on WE, perceived post-war hardships, and symptoms of depression/anxiety (Table 2). Male and female participants significantly differed on SI and depression/anxiety but not on post-war hardships (Table 2).

**Table 1:** **Demographic characteristics and bivariate correlation among variables in the study**

	**Variables**	**Mean**	**SD**	**Minimum-maximum**	**1**	**2**	**3**	**4**	**5**	6
1	Sex (male: n, %)	329, 69			1					
2	Suicidal ideation (n, %)	129, 24			.13**	1				
3	Age at baseline	22.39	2.03	18 - 25	.03 ns	.06 ns	1			
4	Depression/anxiety	21.29	10.47	00 - 54	.26***	-.57***	-.01 ns	1		
5	Post-war hardships	12.47	4.99	00 - 24	-.02 ns	.33***	-.02 ns	.37***	1	
6	General war experiences	41.71	4.19	00 - 52	-.08 ns	.25***	.07 ns	.31***	.63***	1

Key: *SD* = Standard Deviation, *Min* = minimum, *Max* = maximum, *ns* = not significant.

**p < .01; ***p < .001.

Significant statistics are in bold.

**Table 2:** **Descriptive statistics: predictors in the study stratified by suicidal ideation and gender**

**Predictors**	**Suicidal ideation (mean, SD)**	**No suicidal ideation (mean, SD)**	***t*** **-test**
**War experiences**	**45.63 (5.94)**	**40.98 (7.70)**	***t*** **(537) = −5.56,** ***p*** **< .001**
**Perceived post-war hardships**	**11.58 (4.38)**	**15.50 (5.69)**	***t*** **(537) = −7.80,** ***p*** **< .001**
**Symptoms of depression**	**18.01 (8.60)**	**32.05 (8.72)**	***t*** **(537) = −15.67,** ***p*** **< .001**
	**Male (mean, SD)**	**Female (mean, SD)**	***t*** **-test**
War experiences	42.64 (7.31)	41.39 (8.87)	ns
Perceived post-war hardships	6.67 (3.24)	6.75 (3.50)	ns
**Symptoms of depression**	**19.20 (9.91)**	**24.74 (10.49)**	***t*** **(537) = −6.03,** ***p*** **< .001**
**Suicidal ideation**	**.19 (.40)**	**.31 (.46)**	***t*** **(537) = −3.07,** ***p*** **< .001**

Key: *SD* = Standard Deviation, *ns* = not significant.

Significant statistics are in bold.

The results of the univariable analyses of different types of WE on SI are presented in Table 3. “Witnessing violence”, “direct personal harm”, “deaths”, “Involvement in hostilities”, “sexual abuse” and “WE total exposure” significantly predicted SI. The proportion of explained variance for the models for univariable analyses are included and were in the range of R^2^ = 0.02 (*F* (1, 537) = 9.50, *p* < .05) for “involvement in hostilities to R^2^ = 0.06 (*F* (1, 537) = 30.91, *p* < .001) for “WE total exposure” (Table 3).

**Table 3:** **Univariable regression analyses of different types of war experiences on suicidal ideation**

**S/no**	**Types of war experiences**	**β**	**SE**	**95% CI**	**R** ^**2**^	**F - ratio**	***p*** **value**
1	**Witnessing violence**	**0.06**	**0.02**	**0.02 to 0.09**	**R** ^**2**^ **= 0.02**	**(F(1, 537) = 9.55**	***p*** **< 0.05**
2	**Direct personal harm**	**0.06**	**0.02**	**0.02 to 0.09**	**R** ^**2**^ **= 0.02**	**(F(1, 537) = 9.50**	***p*** **< 0.05**
3	Threat to self	0.03	0.02	−0.01 to 0.06	R^2^ = 0.02	(F(1, 537) = 2.03	*ns*
4	**Deaths**	**0.06**	**0.02**	**0.02 to 0.09**	**R** ^**2**^ **= 0.02**	**(F(1, 537) = 9.85**	***p*** **< 0.05**
5	Harm to loved ones	−0.001	0.02	−0.04 to 0.03	R^2^ = 0.001	(F(1, 537) = 0.06	*ns*
6	Material losses	0.02	0.02	−0.02 to 0.05	R^2^ = 0.002	(F(1, 537) = 0.97	*ns*
7	Threat to loved ones	0.05	0.02	−0.02 to 0.09	R^2^ = 0.01	(F(1, 537) = 0.06	*ns*
8	Displacement	0.03	0.02	−0.02 to 0.05	R^2^ = 0.001	(F(1, 537) = 0.49	*ns*
**9**	**Involvement in hostilities**	**0.07**	**0.02**	**0.03 to 0.11**	**R** ^**2**^ **= 0.04**	**(F(1, 537) = 14.07**	***p*** **< 0.001**
10	Separation	0.02	0.02	−0.01 to 0.06	R^2^ = 0.003	(F(1, 537) = 1.61	*ns*
11	**Sexual abuse**	**0.07**	**0.02**	**0.03 to 0.10**	**R** ^**2**^ **= 0.03**	**(F(1, 537) = 13.97**	***p*** **< 0.001**
12	**General War Experiences**	**0.11**	**0.02**	**0.07 to 0.15**	**R** ^**2**^ **= 0.06**	**(F(1, 537) = 30.91**	***p*** **< 0.001**

Key: *R* ^*2*^ = Adjusted R-squared, *SE* = Standard Errors, *CI* = Confidence Intervals, *ns* = not significant, *R* ^*2*^ = Adjusted R^2^, Significant statistics are in bold.

The independent contributions of the types of WE that significantly predicted SI in the univariable analyses (except general exposure to WE) were simultaneously tested in a multivariable regression model. Only “direct personal harm”, (β = 0.04 [95% CI 0.01 to 0.08]) “involvement in hostilities”, (β = 0.04 [95% CI 0.02 to 0.08]) and “sexual abuse” (β = 0.05 [95% CI 0.02 to 0.09]) independently and significantly predicted SI. The proportion of explained variance for the multivariable model was R^2^ = 0.04 (*F* (7, 531) = 5.25, *p* < .001).

**Figure 1:**
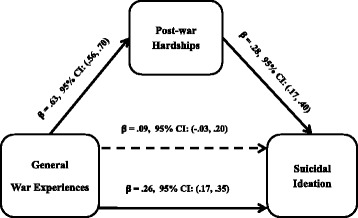
**Mediation by post-war hardships of the relations between past war experiences and suicidal ideation.** Note: The coefficient immediately above the continuous line is before the mediator was added to the model and the one above the dotted line is after the mediator was added to the model. Indirect effect: (mediated effect) = β = .17, 95% CI: (.10, .25). Proportion of total effect = .17/.26 = .65 (or 65%). Ratio of indirect to direct effect = .17/.09 = 1.89. Ratio of total to direct effect = .26/.09 = 2.89. Bootstrap results = β = .78, 95% CI: (.72, .83).

In evaluating the mediation model, there were statistically significant direct associations between WE total exposure and SI (Fgure 1). Post-war hardships were significantly associated with the total number of WE and with SI as well. The association between WE and SI was mediated by post-war hardships by statistically significant indirect path (β = 0.17 [95% CI 0.12 to 0.22]) accounting for approximately 69% of the effect of WE on SI. The effects of WE total exposure on SI reduced and was no longer statistically significant after including post-war hardships (β = 0.09 [95% CI: −0.03 to 0.20]) indicating complete mediation. The proportion of explained variance for the model with only WE was R^2^ = 0.06 (*F* (1, 537) = 30.91, *p* < .001) but this increased to R^2^ = 0.11 (*F* (2, 536) = 28.42, *p* < .001) after adding post-war hardships in the mediation model (Figure 1).

**Figure 2:**
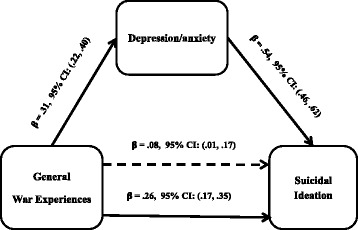
**Mediation by symptoms of depression/anxiety of the relations between past war experiences and suicidal ideation.** *Note*: The coefficient immediately above the continuous line is before the mediator was added to the model and the one above the dotted line is after the mediator was added to the model. Indirect effect: (mediated effect) = β = .18, 95% CI: (.11, .22). Proportion of total effect = .18/.26 = .69 (or 69%). Ratio of indirect to direct effect = .18/.08 = 2.25. Ratio of total to direct effect = .26/.09 = 2.25. Bootstrap results = β = .88, 95% CI: (.82, .96).

Finally, in the mediation model with symptoms of depression/anxiety, there were statistically significant direct associations between general exposure to WE and SI (Figure 2). Symptoms of depression/anxiety were significantly associated with general exposure to WE and with SI as well. The association between WE and SI was mediated by symptoms of depression/anxiety by statistically significant indirect path (β = 0.18 (95% CI 0.12 to 0.22)) accounting for approximately 65% of the effect of WE on SI. The effects of WE on SI reduced but remained statistically significant after including symptoms of depression (β = 0.08 (95% CI: 0.01 to 0.17)) indicating partial mediation. The proportion of explained variance for the model with only WE was R^2^ = 0.06 (*F* (1, 537) = 30.91, *p* < .001) but this increased to R^2^ = 0.33 (*F* (2, 536) = 107.78, *p* < .001) after adding symptoms of depression/anxiety in the mediation model. When the analysis was performed without the item on SI in the depression/anxiety scale, the results were similar with approximately 64% of the effect of WE on SI mediated by symptoms of depression/anxiety. Finally, in this study, the VIF were all less than 3.0, indicating that the results were not affected by multi-collinearity.

## Discussion

Suicide is common in the aftermath of war and empirical research to inform interventions are lacking. The current study assessed the contributions of different types of previous WE in predicting current SI and assessed the mediating roles of post-war hardships and depression/anxiety in the relations between WE and SI in FCS in Northern Uganda. Witnessing violence, ddirect personal harm, deaths, Involvement in hostilities, sexual abuse, and exposure to previous WE significantly predicted current SI and direct personal harm, involvement in hostilities and sexual abuse independently predicted current SI when assessed in a multivariable model only. While testing the mediation hypothesis that post-war hardships and depression significantly accounted for the association between previous WE and current SI, the proportion of the total effects mediated by post-war hardships were 69% and 65% for depression/anxiety. The relations between previous WE and current SI was no longer significant after including post-war hardships. For symptoms of depression/anxiety, the effects of previous WE on current SI attenuated but remained significant.

### Meaning and implications of the findings

The effects of specific WE on SI are noteworthy. The FCS were also involved in combat and used as human shields during battles with government forces where many lost their lives or sustained serious injuries [15-17,45,46]. Female FCS were deliberately abducted and forcefully married to combatants while in rebel captivity [45]. Experiences of direct personal harm, involvement in hostilities and sexual abuse may all elicit strong feelings of shame, an emotion linked to SI [47]. As such, it is entirely consistent that personal harm, involvement in hostilities, and sexual abuse are risk factors for current SI. Health practitioners should be cognizant of the effects of different types of previous WE on FCS to plan effective interventions. Understanding that certain WE contribute to SI is clinically important, but the small effect size (i.e. 4% of variance accounted for by the three forms of WE) also needs to be borne in mind.

The aftermath of war is characterised by numerous stressors and hardships [48-51]. This might explain why post-war hardships accounted for nearly 70% of the relations between WE and SI and why the effects of previous WE on current SI ceased to be significant after post-war hardships were included in the regression model. These hardships and difficulties may include poverty, unemployment, lack of essentials, and inability to access health services for mental and physical health problems associated with their previous WE [52]. The findings presented here are consistent with results of previous studies indicating that post-war hardships, [49] family and community violence, [50,51,53,54] and stigma and discrimination, [55,56] are risk factors for persistent mental health problems such as PTSD, depression, and alcohol and drug abuse, all of which are linked to current SI [32,36-39,57]. Mediation analyses demonstrated that both post-war hardships and depression/anxiety could act as pathways by which previous WE impacts on current SI. Therefore, interventions to mitigate the effects of previous WE on current SI could consider reducing both post-war hardships and treating depression/anxiety.

### Strengths and limitation

The current study has a number of strengths. First, many studies were conducted at the height of the war with the possibility of the findings being contaminated with on-going incidents [58]. The current study was carried out six years after the war ceased and the FCS were reintegrated into their communities. The findings of this study are therefore not influenced by on-going WE. Second, in contrast to previous research in low resource settings, the sample size of this study is comparatively larger making the results more credible [56,59]. Finally, the instruments used to assess WE and depression/anxiety were adapted and normed in similar war-affected populations before [17].

However, our findings should be considered in the context of some limitations. First, the use of a cross-sectional design limits causal inferences. Second, the retrospective report of previous WE is prone to recall bias. Participants who experience mental health problems as a result of the war are known to perceive more severe life stressors [60]. To reduce this possible source of bias related to over- or under-reporting of previous WE in our study, we refrained from using severity scores but instead used the number (counts) of previous WE in computing the scores for WE. Third, due to multiple analyses of different types of war events, chance findings are possible. However, in both mediation analyses, the general war exposure consisted of all the 52 items in the war events questionnaire. Likewise, in this study, the VIF were all less than 3.0, indicating that multi-collinearity was not a problem. Finally, potentially important variables for determining suicide such as social support were not assessed at baseline and follow-up. These important variables will be assessed in the next wave of data collection.

### Implications of the findings

Policies to reduce suicidal behaviours should aim to reduce the burden of post-war hardships and treat depression that keep FCS in the path of further mental health problems and feelings of hopelessness leading to SI. Easing post-war hardships by training in skills that increases opportunities for employment to enable FCS to reconstruct their lives may reduce the hopelessness that leads to suicidal behaviours. Health programs should take into considerations certain types of previous WE such as direct personal harm and sexual abuse when treating FCS. Further research should be conducted on the mechanisms through which WE are linked to suicidal behaviours to illuminate and inform interventions. The roles of factors such as stigma and discrimination, social support networks, and coping strategies may provide windows of opportunities for interventions and should be further explored.

## Conclusions

Specific types of WE were risk factors for SI. In addition, the current study showed that post-war hardships and depression/anxiety are key determinants of the relations between previous WE and current SI in FCS. Notwithstanding the mediating roles of post-war hardships and depression/anxiety, other factors may be associated with individual differences in SI among FCS. Therefore, to improve undesirable effects of previous WE on current SI among FCS, interventions to reduce post-war hardships and depression/anxiety among FCS and other war-affected populations should be considered.
